# Ultrasensitive Surface Plasmon Resonance Biosensor Using Blue Phosphorus–Graphene Architecture

**DOI:** 10.3390/s20113326

**Published:** 2020-06-11

**Authors:** Keyi Li, Lintong Li, Nanlin Xu, Xiao Peng, Yingxin Zhou, Yufeng Yuan, Jun Song, Junle Qu

**Affiliations:** Key Laboratory of Optoelectronic Devices and Systems of Ministry of Education and Guangdong Province, College of Physics and Optoelectronic Engineering, Shenzhen University, Shenzhen 518060, China; keyi_li07070023@163.com (K.L.); lintong_li@163.com (L.L.); nanlim_xu@163.com (N.X.); pengxiao_px@szu.edu.cn (X.P.); yls772@163.com (Y.Z.); songjun@szu.edu.cn (J.S.); jlqu@szu.edu.cn (J.Q.)

**Keywords:** surface plasmon resonance biosensor, ultrasensitive detection, blue phosphorus-graphene architecture, phase modulation

## Abstract

This study theoretically proposed a novel surface plasmon resonance biosensor by incorporating emerging two dimensional material blue phosphorus and graphene layers with plasmonic gold film. The excellent performances employed for biosensing can be realized by accurately tuning the thickness of gold film and the number of blue phosphorus interlayer. Our proposed plasmonic biosensor architecture designed by phase modulation is much superior to angular modulation, providing 4 orders of magnitude sensitivity enhancement. In addition, the optimized stacked configuration is 42 nm Au film/2-layer blue phosphorus /4-layer graphene, which can produce the sharpest differential phase of 176.7661 degrees and darkest minimum reflectivity as low as 5.3787 × 10^−6^. For a tiny variation in local refractive index of 0.0012 RIU (RIU, refractive index unit) due to the binding interactions of aromatic biomolecules, our proposed biosensor can provide an ultrahigh detection sensitivity up to 1.4731 × 10^5^ °/RIU, highly promising for performing ultrasensitive biosensing application.

## 1. Introduction

Since the discovery of black phosphorus (BP), another two dimensional (2D) allotrope of phosphorus named blue phosphorus (BlueP) has been predicted theoretically and successfully engineered in laboratory [1,2,3,4,5]. Unlike black phosphorus, the phosphorus atoms in the BlueP crystal lattice are arranged in a lower buckled honeycomb. However, blue phosphorus has shown high stability with the previously reported black phosphorus [2]. In addition, theoretical studies have shown that blue phosphorus has a tunable band gap ranging from 2 eV to 1.2 eV from monolayer to bulk [1], making it a promising candidate for designing novel optoelectronic devices such as gas sensors [6] quantum spin Hall insulators [7] and Dirac cones [8]. 

Recently, efficiently integrating various 2D materials to form versatile Van der Waals heterostructures is an important strategy to produce a host of unprecedented physical and electric properties of 2D material, due to the mutual Van Der Waals interactions in stacked configuration [9,10]. Compared with a single 2D material, the heterostructures can generate more novel electronic and optical features such as phonon frequency, binding energy, and carrier mobility [11]. Actually, the optical and electronic features of 2D heterostructures are highly dependent on both the number of 2D material layers and the stacking patterns. Due to their excellent sensitivity enhancement effect, 2D material-based plasmonic sensors have been employed to perform practical biosensing [12,13,14,15,16]. To date, layered blue phosphorus is an emerging semiconducting material, which has been employed in designing novel plasmonic sensors. For example, surface plasmon resonance (SPR) heterostructures composed of blue phosphorus and molybdenum disulfide (MoS_2_) can provide a significant sensitivity enhancement compared with conventional noble metal film-based SPR sensors [17,18]. More recently, Yue et al. [19] proposed a novel SPR biosensor designed by a Ag–Au bimetallic film coated with blue phosphorus, transition metal dichalcogenides (TMDCs), and graphene layers. Using the bilayer BlueP/WS_2_, the proposed configuration can produce a sensitivity as high as 335.4 °/RIU. Although these above observations have shown good sensitivity enhancement using BlueP nanosheets, these hybrid plasmonic architectures designed by angular modulation cannot meet the demand for monitoring the biological molecules in an ultralow concentration. Thus, it is of considerable significance to develop novel BlueP-enhanced plasmonic biosensors with ultrahigh detection sensitivity.

Working as an excellent plasmonic material, graphene has been widely integrated with other 2D materials (such as transition metal dichalcogenides [20], black phosphorus [21], and MXenes [22]), producing a significant detection sensitivity. In addition, graphene is a long-term oxidation barrier, which can generate effective antioxidation. Herein, we theoretically proposed a novel ultrasensitive SPR biosensor consisting of plasmonic gold film, few layers of blue phosphorus, and a graphene layer. Remarkably, our proposed architecture designed by phase modulation is more superior to conventional angular modulation, producing four orders of magnitude for sensitivity enhancement. The simulation results showed that the best configuration employed for generating the strongest SPR excitation is a 42 nm Au film/2-layer BlueP/4-layer graphene. More importantly, our proposed biosensor can provide a phase detection sensitivity as high as 1.4731 × 10^5^ °/RIU, which is enhanced by almost 440 times compared with the previously reported BlueP-based SPR sensor (335.4 °/RIU) [18]. We believe that our proposed architecture is a promising candidate for exploring novel phosphorene-based sensors. 

## 2. Methodology

**Figure 1:**
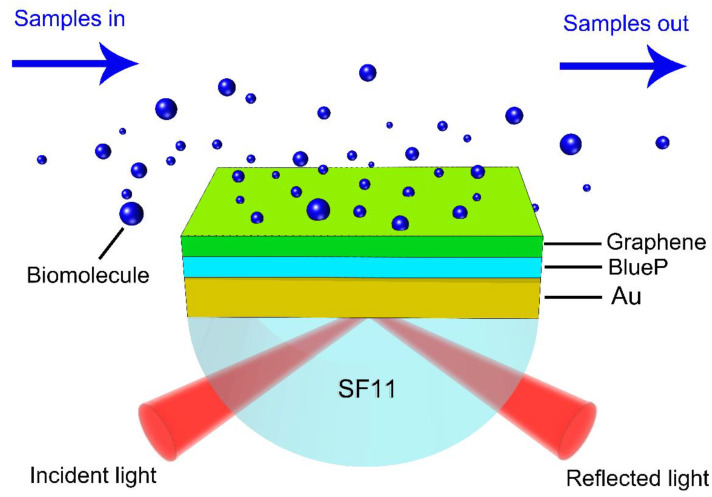
Schematic diagram of the SF11 prism/Au film/ BlueP/graphene biosensor.

The proposed SPR biosensor is composed of a SF11 prism, plasmonic Au thin film, few layers of blue phosphorus, and a graphene layer, as shown in Figure 1. In this study, we chose a classical Kretschmann configuration employed for SPR excitation to study the sensitivity enhancement performance. A p-polarized incident light at 632.8 nm was employed to excite the SPR enhancement in the gold film–BlueP–graphene heterostructure. With the help of refractive index matching liquid, the Au thin film was well integrated with a hemispherical SF11 prism. After that, the BlueP interlayer was stacked onto the top of the gold film. Next, a few layers of graphene were deposited onto the BlueP interlayer to prevent the BlueP from oxidation. Moreover, the graphene overlayer will not only enhance the biosensing ability, but can also significantly capture aromatic biomolecules via non-covalent π–π stacking interaction force [23,24,25]. Therefore, there is no need for further surface modification for the BlueP–graphene interface. The top layer is a sample cell filled with liquid solvent. The fluid containing aromatic biological molecules have close contact with the graphene overlayer, resulting in the adsorption behavior of biological molecules. It is worth noting that when the targeted aromatic biomolecules are successfully adsorbed from the sample medium onto the graphene surface via π–π stacking interaction force, there is a variation in local refractive index of the sensing interface, generating a perturbation for an enhanced electric field. Ultimately, the perturbation can result in a change in the surface plasmon wave vector, which can be monitored by measuring SPR reflectivity and phase.

Similar to SPR reflectance, both reflective phantom interface (RPI) and interferometric reflectance imaging (IRI) techniques are low-cost, operational simplicity, and label-free optical approaches for monitoring analytes [26,27,28]. The signal transduction of both RPI and IRI is mainly based on spectral reflectivity. However, there are four approaches employed for measuring SPR signal: intensity modulation, angle modulation, wavelength modulation, and phase modulation. Intensity modulation is the simplest method for directly measuring the reflected light intensity. However, the SPR signal usually suffers from the noise of excitation light. Angle modulation is the most widely used approach in prism-based SPR configuration. Through tuning various incident angles, the coupling strength of SPR can be modulated. Compared with the intensity modulation, the angle modulation can provide a higher detection sensitivity. In addition, the working principle of wavelength modulation is that with a fixed incident angle, a polychromatic light with multiple frequencies is employed to excite the SPR effect and the reflected intensity light is measured. Finally, the working principle of phase modulation is to extract the phase information on reflected light. Under SPR conditions, the phase of reflected light can produce a sharp jump behavior, which usually occurs at the SPR dip with minimum reflectivity. It is worth noting that phase modulation introduces two light beams, namely a probe beam and a reference beam. Due to their interference, the differential phase between p-polarized light and s-polarized light can be observed. Compared with the intensity, angle or wavelength modulation, the phase modulation is the most sensitive, showing great potential in designing ultrasensitive SPR biosensors.

Prior to phase modulation study, the refractive index of each layer in our proposed configuration is required. Under the illumination of 632.8 nm incident light, the refractive index of the SF11 prism, plasmonic gold film, BlueP interlayer, graphene overlayer, and sensing medium (deionized water) was determined to be 1.7786 [29], 0.1838 + i 3.4313 [20], 2.1666 + i 0.1005 [30], 3.0000 + i 1.1487 [31], and 1.3330 [29], respectively. In addition, the thickness of the monolayer of blue phosphorus and graphene was 0.123 nm [30], and 0.34 nm [31], respectively. 

Next, both the transfer matrix method and Fresnel equations were employed to study the plasmonic biosensing performance in an N-layered stacked SPR model [21]. Through calculating the SPR reflectivity and phase, the differential phase can be extracted. In the N-layered configuration, all the stacked materials are considered as optically isotropic and non-magnetic. For the phase modulation method, p-polarized (TM) light is employed to excite and produce the SPR signals in the planar metal–dielectric interface, while s-polarized (TE) light can be used as a reference signal to suppress the environmental noise [32]. As a result, the differential phase (φd) between TM light and TE light can be calculated by Equation (1).

(1) φ=d|φp−φs|

The phase of p-polarized light ($\varphi_{p}$) can be extracted by Equation (2)

(2) $$\varphi_{p}=\arg(r_{p})$$

where $r_{p}$ is the reflection coefficient. According to the Fresnel Equation and Snell’s law, the reflection coefficient $r_{p}$ can be described as Equation (3)

(3) $$r_{p}=\frac{E_{rp}}{E_{ip}}=\frac{n_{t}\cos\theta_{i}-n_{i}\cos\theta_{t}}{n_{t}\cos\theta_{i}+n_{i}\cos\theta_{t}}\;(\mathrm{Fresnel}\;\mathrm{Equation});\;n_{i}\sin\theta_{i}=n_{t}\sin\theta_{t}\;(\mathrm{Snell}’s\;\mathrm{law})\;$$

where $n_{t}$ and $n_{i}$ are the refractive index of two different mediums at the interface, respectively.

For the N-layered model, the total reflection coefficient $r_{p}$ can be calculated by the transfer matrix method. The matrix can be defined as *M*, where *k* stands for the *k*-th layer [33]:

(4) $$M=\prod_{k=2}^{N-1}M_{k}=\left[\begin{array}{cc} M_{11} & M_{12}\\ M_{21} & M_{22} \end{array}\right]\;;\;M_{k}=\left[\begin{array}{cc} \cos\beta_{k} & -i\frac{\sin\theta_{k}}{q_{k}}\\ -iq_{k}\sin\beta_{k} & \cos\beta_{k} \end{array}\right]$$

where qk=(εk−nSF112sin2θ1)12εk, $\beta_{k}=\frac{2\pi d_{k}\lambda }{\lambda }{\left(\varepsilon_{k}-n_{SF11}^{2}\sin^{2}\theta_{1}\right)}^{1/2}$, and $\theta_{1}$ is the incident angle at the first layer. $\varepsilon_{k}$ and $d_{k}$ are the optical constant and the thickness of *k*-th layer, respectively. The first boundary of the tangential fields is defined as Z_1_ = 0, and the last boundary Z_*N*−1_ is given by

(5) $$\left[\frac{U_{1}}{V_{1}}\right]=M\left[\frac{U_{N-1}}{V_{N-1}}\right]$$

where *U* stands for the tangential components of the electric fields at the interface, while *V* shows those in the magnetic fields [34]. 

Finally, the complex reflection coefficient $r_{p}$ of the N-layer for the p-polarized light can be defined as

(6) $$r_{p}=\frac{\left(M_{11}+M_{12}q_{N}\right)q_{1}-\left(M_{21}+M_{22}q_{N}\right)}{\left(M_{11}+M_{12}q_{N}\right)q_{1}+\left(M_{21}+M_{22}q_{N}\right)}$$

With the help of $r_{p}$, the reflectivity of SPR curve (RP) can be calculated by Equation (7).

(7) R=P|rp|2

It is worth noting that the above stated equations are also applicable, except for parameter $q_{k}$ for s-polarized light.

(8) qk=(εk−nSF112sin2θ1)12

In this study, we determined the SPR sensing performance of our proposed configuration by employing the two parameters of phase detection sensitivity and enhancement factor (EF). The phase detection sensitivity ($S_{Au-BlueP-graphene}$) of our proposed model is determined by Equation (9)

(9) $$S_{Au-BlueP-graphene}=\frac{\Delta \varphi_{Au-BlueP-graphene}}{\Delta n_{bio}}$$

where $\Delta \varphi_{Au-BlueP-graphene}$ stands for the change in differential phase, and $\Delta n_{bio}$ denotes the change in local refractive index due to the binding interactions between the sensing interface and targeted aromatic biomolecules.

In addition, the enhancement factor (EF) of phase detection sensitivity with Au film–BlueP–graphene versus that of the bare gold thin film can be given as Equation (10)

(10) EF=SAu−BlueP−grapheneSAu=ΔφAu−BlueP−grapheneΔnbioΔφAuΔnbio=ΔφAu−BlueP−grapheneΔφAu

## 3. Results and Discussion

**Figure 2:**
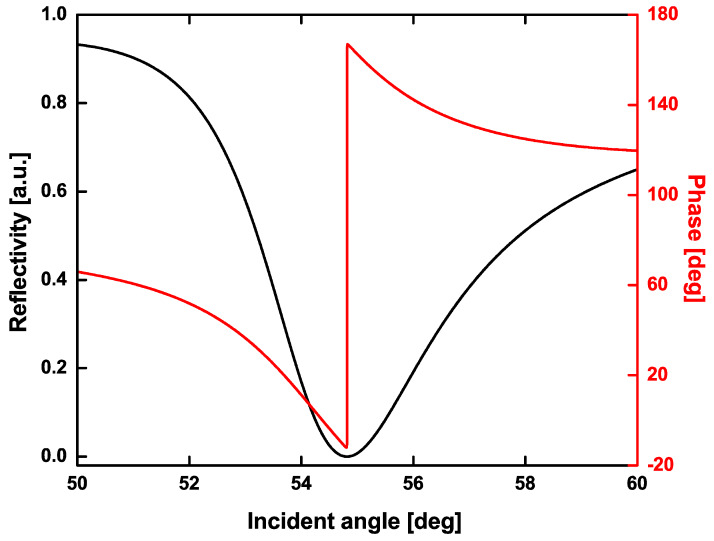
The reflectivity (black) and phase (red) variation with respect to the incident angle. The number of BlueP and graphene layers is 2 and 4, respectively. The Au film thickness is 42 nm. The incident wavelength is 632.8 nm.

Using the transfer matrix theory and Fresnel equations, both the reflectance and phase transition were systematically studied. The relationship between reflectivity and the incident angle was plotted in a SPR curve, where the dip of SPR curve is the minimum reflectivity. In addition, the sharp phase jump usually occurs at the dip of the SPR curve as the intensity of reflected light is close to zero. It can be found that both the reflectivity and phase transition can be monitored by changing the thickness of the plasmonic Au film and the number of blue phosphorus and graphene layers. Employing deionized water (n_water_ = 1.3330) as the sensing solvent, the best stacked configuration was obtained, as shown in Figure 2. The heterostructures consisting of 42 nm Au film, 2-layer BlueP, and 4-layer graphene can generate an ultralow minimum reflectivity as low as 5.3787 × 10^−6^ (black curve, Figure 2) when the incident angle is located at 54.8152°. This demonstrates that the strongest SPR excitation can be realized as the incident angle is fixed at 54.8152°. More importantly, there is a significant phase jump (red curve, Figure 2) at the point of 54.8152°, which is quite suitable for performing phase modulation study.

**Figure 3:**
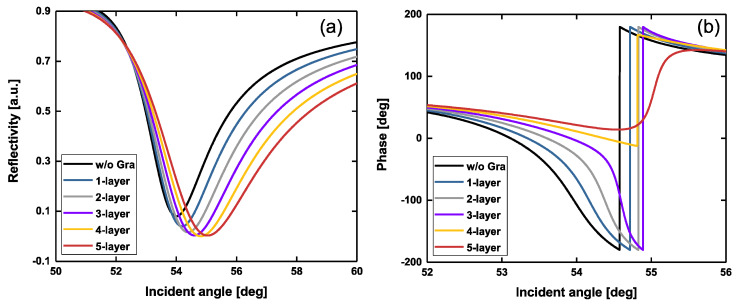
Variation of reflectivity (**a**) and phase (**b**) with respect to the incident angle by tuning the number of graphene overlayers ranging from 0 to 5 layers. The Au film thickness is 42 nm. The number of blue phosphorus layers is 2. The incident wavelength is 632.8 nm.

Next, we studied the effects of graphene layer introduction on the reflectivity and phase jump. For a fixed thickness Au film of 42 nm and 2-layer blue phosphorus, the variations in both phase and reflectivity were calculated by tuning the number of graphene layers ranging from 0 to 5, as shown in Figure 3. 

The simulation results showed that the introduction of graphene ranging from 0 to 4 layers could significantly improve the reflectivity and phase in our proposed configuration. In the absence of a graphene layer, the 42 nm Au film/2-layer BlueP configuration had a reflectivity of 3.6543 × 10^−5^. However, the 42 nm Au film/2-layer BlueP/4-layer graphene configuration had a lower reflectivity, as low as 5.3787 × 10^−6^. It has been reported that monolayer graphene can absorb 2.3% of incident photons, converting this into energy [35]. Thus, the addition of 4-layer graphene can theoretically increase 9.2% of photon absorption. However, the ultralow reflectivity of 5.3787 × 10^−6^ indicates that our proposed biosensor is not a simple stacked configuration. Due to the strong coherent optical absorption in multiple graphene layers [36], ultralow optical reflectivity could be generated. With the help of graphene, the phase jump becomes steep. However, excessive graphene layers can also generate negative influences on both the reflectivity and phase jump, as shown in Figure 3. When the 5-layer graphene was introduced, the reflectivity (red curve, Figure 3a) became larger. Additionally, the phase jump (red curve, Figure 3b) became slower. The reason is that the energy loss generated by the 5-layer graphene is more than photon absorption, breaking the balance between photon absorption and energy loss.

**Figure 4:**
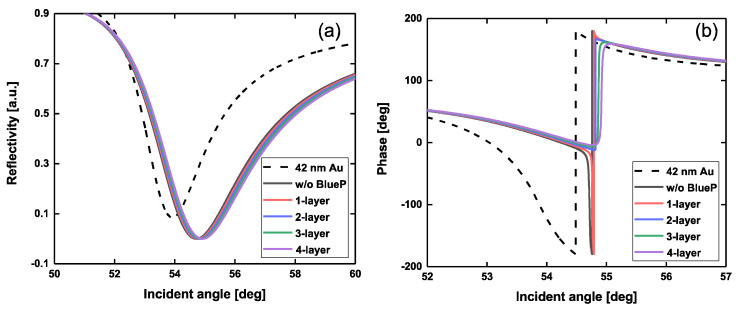
Variation of reflectivity (**a**) and phase (**b**) with respect to the incident angle by changing the number of BlueP interlayers ranging from 0 to 4 layers. The Au film thickness is 42 nm. The number of graphene is 4. The incident wavelength is 632.8 nm. Note: the black dotted curve in Figure 4a stands for the SPR reflectance originating from the 42 nm Au film. The black dotted curve in Figure 4b denotes the SPR phase originating from the 42 nm Au film.

When both the thickness of the Au film and graphene was fixed, we also calculated the reflectivity and phase by varying the number of BlueP layers, as shown in Figure 4. Without the BlueP and graphene layers, the minimum reflectivity of 42 nm gold film was 0.0842. This means that only the pure gold film cannot support strong SPR enhancement due to the poor efficiency of photon absorption. However, with the help of 4-layer graphene, the efficiency of photon absorption can be significantly developed, producing a minimum reflectivity of 3.6543 × 10^−5^. By introducing a 2-layer BlueP interlayer, the minimum reflectivity can be further reduced to 5.3787 × 10^−6^, reducing it by almost seven times. Meanwhile, the phase change also showed the steepest transition due to the addition of 2-layer BlueP.

**Figure 5:**
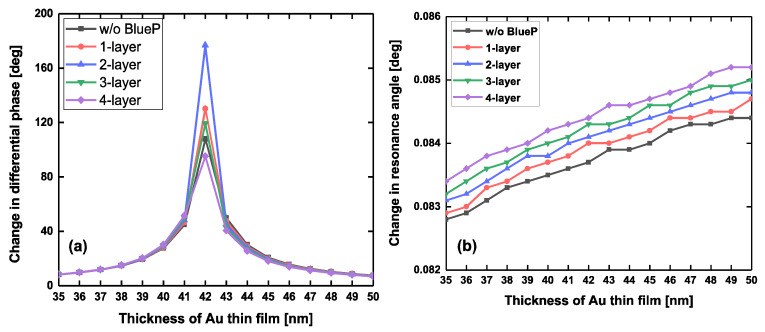
For a tiny refractive-index variation as small as 0.0012 RIU approaching the sensing interface, the change in differential phase (**a**), red-shifts based on SPR incident angle (**b**) with respect to the thickness of gold film ranging from 35 to 50 nm. The excitation wavelength is 632.8 nm.

To compare with the biosensing performance based on our proposed biosensor, we calculated the changes in both differential phase (Δφd) and SPR angle (△*θ_SPR_*) for a tiny refractive index variation (∆n = 0.0012, corresponding to detect less than 8 kDa biomolecules as low as 1 pM [20]) approaching the sensing interface, as shown in Figure 5 and Table 1. It can be found that as the Au film thickness was smaller than 42 nm, the differential phase (Δφd) showed a positive response for the addition of blue phosphorus interlayers. Conversely, as the gold film thickness was larger than 42 nm, the differential phase decreased sharply. The largest differential phase was 176.7661°, corresponding to 42 nm Au film, 2-layer blue phosphorus, and 4-layer graphene, respectively. In addition, Figure 5b shows that the largest red-shift based on the SPR angle was 0.0837°, 0.0844°, 0.0847°, 0.0848°, 0.0850°, 0.0852°, which corresponds to the 0, 1, 2, 3, 4 layers of blue phosphorus, respectively. Undoubtedly, these tiny red-shifts created by angle modulation cannot be considered a readout using an experimental SPR instrument. Instead, the largest differential phase can work as an important indicator for monitoring the change in the refractive index of the sensing interface. Moreover, an ultrahigh detection sensitivity of 1.4731 × 10^5^ °/RIU can provide an enhancement factor of four orders of magnitude, compared to the tiny red-shifts in SPR angle. To achieve the ultrasensitive biosensing, the best configuration of 42 nm Au film/2-layer blue phosphorus/4-layer graphene is preferred. We also studied the changes in phase detection sensitivity by slightly varying the incident angle in an increment of ±0.01 degrees, as shown in Table S1 (Supplementary Materials). As the incident angle was smaller than 54.8152°, the obtained phase detection sensitivity from 42 nm Au film/2-layer blue phosphorus/4-layer graphene decreased sharply. However, when the incident angle was no more than 54.8852°, the proposed configuration 42 nm Au film/2-layer blue phosphorus/4-layer graphene still showed ultrahigh detection sensitivity. Experimentally, the practical incident angle should be positively shifted away from the optimized SPR angle of 54.8152° with an increment of 0.01–0.07 degrees.

**Figure 6:**
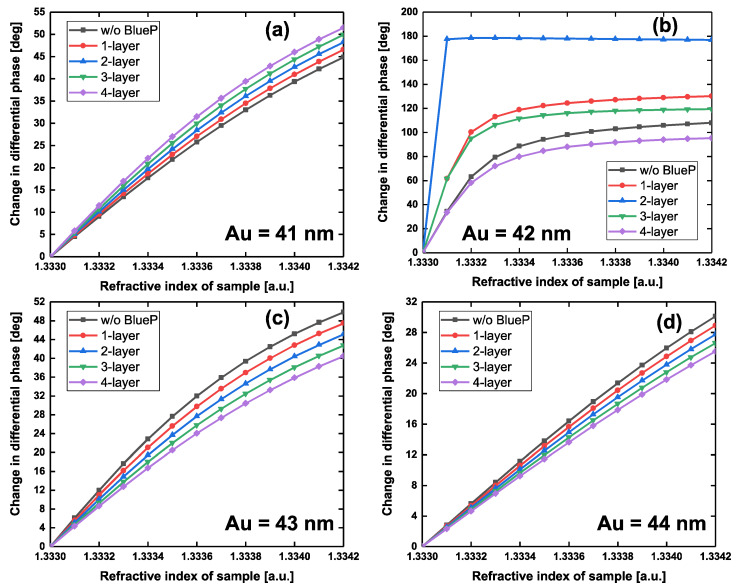
Change in differential phase with respect to the variation in local refractive index of the sensing interface by modulating the numbers of the BlueP interlayer and the thickness of Au film: (**a**) 41 nm, (**b**) 42 nm, (**c**) 43 nm, and (**d**) 44 nm, respectively.

In addition, we also investigated the change in differential phase with respect to the refractive index ranging from 1.3330 to 1.3342 RIU by modulating the thickness of Au film from 40–45 nm and the number of BlueP interlayers, as shown in Figure 6 and Figure S1 (Supplementary Materials). When the thickness of the Au film was less than 41 nm, the changes in differential phase indicated an almost linear relationship with the increase in the refractive index of the sample. Additionally, there was no significant response in change in the differential phase with the introduction of the BlueP interlayer. It is worth noting that there were significant changes in the differential phase when the thickness of Au film was 42 nm. Moreover, the addition of the BlueP layer is very sensitive in generating steep changes in differential phase. For example, the steepest change in differential phase (blue curve, Figure 6b) was obtained by such a stacked configuration: 42 nm gold film deposited with 2-layer blue phosphorus and 4-layer graphene. However, when the thickness of Au film thickness was no less than 43 nm, the addition of blue phosphorus was useless in producing sensitivity enhancement due to excessive energy loss.

**Figure 7:**
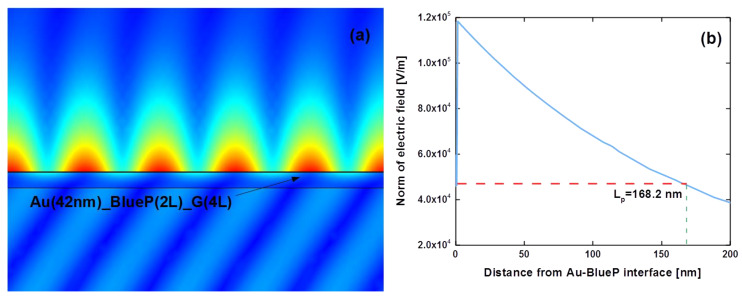
(**a**) The enhanced electric field distribution approaching the sensing interface based on the 42 nm Au film/2-layer BlueP/4-layer graphene. (**b**) Evanescent decay of significantly enhanced electric field penetrating into the sensing medium. The red line shows that the penetration depth (LP) is ∼168.2 nm, which can be obtained by calculating the distance from the point with the largest intensity to the point whose intensity reduces to 1/e of the largest value. Annotation: Au (42 nm)_BlueP (2L)_G(4L) in Figure 7a is the abbreviation of our proposed configuration (42 nm Au film/2-layer BlueP/4-layer graphene.

To further verify the strongest SPR excitation in the 42 nm Au film/2-layer BlueP/4-layer graphene configuration, finite element analysis theory was employed to study the enhanced electric field distribution approaching the BlueP–graphene sensing interface, as shown in Figure 7. Under the illumination of p-polarized light at 632.8 nm, a significantly enhanced electric field (Figure 7a) was generated at the sensing interface of BlueP-graphene. Additionally, the electric field is regularly distributed in our proposed configuration. Along the direction of sensing medium water, the enhanced electric field showed an obvious change in exponential decay with a penetration depth (Lp) of 168.2 nm. Thus, the highly enhanced electric field can contribute to the ultrahigh detection sensitivity of 1.4731 × 10^5^ °/RIU, responding to a tiny refractive index variation in sensing interface.

**Figure 8:**
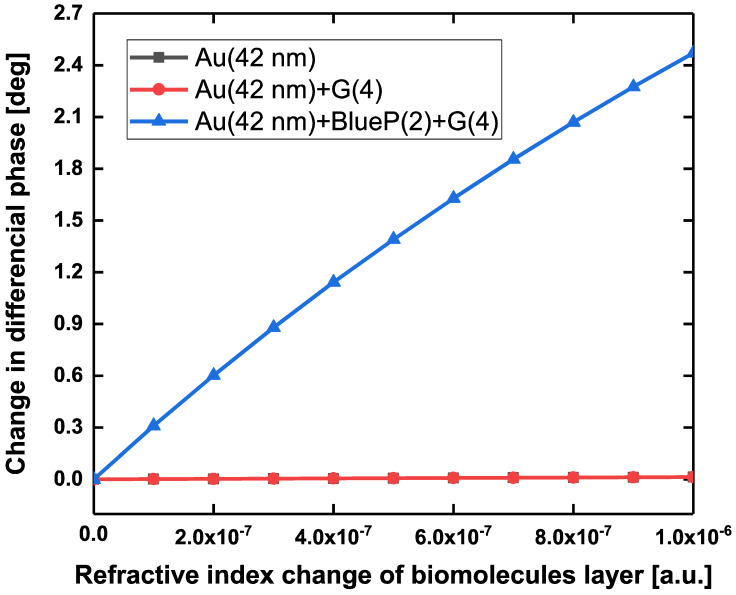
For an extremely tiny refractive index variation in a level of 1 × 10^−6^ RIU, the comparison of change in differential phase with regard to the pure 42 nm Au film, 42 nm Au film/4-layer graphene, and 42 nm Au film/2-layer BlueP/4-layer graphene. Annotation: Au (42 nm)_BlueP (2L)_G(4L) in Figure 8 is the abbreviation of our proposed configuration (42 nm Au film/2-layer BlueP/4-layer graphene.

To determine the enhancement factor of phase sensitivity, the linear signal response based on the change in differential phase was plotted with respect to the variation in the refractive index of the sensing interface as low as 10^−6^ RIU. Figure 8 shows our proposed SPR biosensor configuration still had a positive response for an extremely tiny refractive index change as low as 10^−6^ RIU. Compared with other SPR configurations such as the pure 42 nm Au film and the 42 nm Au film deposited with 4-layer graphene, the enhancement factor (EF) originating from our proposed biosensor configuration was almost 250-fold. Typically, as the concentration of targeted analytes gradually goes down to a single molecule level, the weak biomolecular binding interaction would produce a very tiny perturbation for the local refractive index of the sensing interface. If the variation in local refractive index is perceived by the existing surface plasmon waves in the sensing interface, the enlarged SPR signal response (differential phase) can be obtained due to the wave vector matching condition.

Prior to this work, numerous theoretical SPR configurations have shown their feasibility for performing plasmonic biosensing. Here, our proposed configuration shows the feasibility of 2D material blue phosphorus employed for sensitivity enhancement. For a comparison with our work, we summarized other 2D material-enhanced SPR models, as shown in Table 2. For these SPR sensors, both the angular sensitivity and phase sensitivity were further developed by introducing 2D material nanosheets. Under the excitation of 632.8 nm, our proposed model of Au–BlueP–graphene is more advantageous when compared to other previously proposed SPR configurations. It is worth noting that our proposed configuration is in idealized mode for monitoring aromatic biomolecules. However, specificity detection is more promising in practical application. To perform specificity detection using the blue phosphorus–graphene architecture, graphene surface functionalization is indispensable, largely by changing the thickness of the graphene overlayer. Although graphene functionalization may reduce the phase detection sensitivity, specificity detection can be achieved. In a way, a practical SPR biosensor should have multiple balanced advantages.

## 4. Conclusions

In this study, we proposed a novel SPR biosensor with ultrahigh detection sensitivity by integrating emerging blue phosphorus and graphene nanosheets onto plasmonic gold film. Both light absorption and energy loss in our proposed configuration can be maintained in a balance state by tuning the thickness of the gold film and the number of blue phosphorus interlayers. In addition, our proposed biosensor is created by phase modulation, providing four orders of magnitude sensitivity enhancement compared with angular modulation. In addition, the optimized configuration can produce the sharpest change in differential phase (176.7661 degrees) and darkest minimum reflectivity (5.3787 × 10^−6^). More importantly, the highest detection sensitivity of 1.4731 × 10^5^ °/RIU can be obtained using the following optimized configuration: 42 nm Au film coated with 2-layer blue phosphorus, and 4-layer graphene. Compared with the pure 42 nm Au film and 42 nm Au film deposited with 4-layer graphene, the linear phase sensitivity was enhanced by almost 250 times. It can be expected that our proposed biosensor is a promising candidate for designing BlueP-based biosensors for monitoring biomolecules in an ultralow concentration.

## Figures and Tables

**Table 1:** The optimized values of Au film thickness, the number of BlueP layers, the changes in SPR incident angle (△*θ_SPR_*), the changes in differential phase (Δφd), minimum reflectivity, and sensitivity.

Au Film	Number of BlueP	*θ_SPR_*	$\Delta \theta_{SPR}$	Δφd	Minimum Reflectivity	Sensitivity (°/RIU)
42 nm	0	54.7152°	0.0837°	107.9544°	3.6543 × 10^−5^	8.9962 × 10^4^ °/RIU
42 nm	1	54.7650°	0.0840°	130.1169°	1.3667 × 10^−5^	1.0843 × 10^5^ °/RIU
42 nm	2	54.8152°	0.0841°	176.7661°	5.3787 × 10^−6^	1.4731 × 10^5^ °/RIU
42 nm	3	54.8658°	0.0843°	119.2825°	1.1914 × 10^−5^	9.9402 × 10^4^ °/RIU
42 nm	4	54.9167°	0.0844°	95.1485°	3.3275 × 10^−5^	7.9290 × 10^4^ °/RIU

**Table 2:** Comparison of detection sensitivity calculated by angular or phase modulation based on previously reported work.

SPR Configuration	Prism	Incident Wavelength (nm)	Angular Sensitivity (°/RIU)	Phase Sensitivity (°/RIU)	References
**Au–Si–WS_2_**	SF10	632.8	147.88	----------	[37]
**Au–BlueP/MoS_2_–Graphene**	BK7	632.8	204	----------	[38]
**Ag–BlueP/MoS_2_**	CaF_2_	662	432.15	----------	[17]
**Au–Silicon–BlueP/MoS_2_**	BK7	632.8	230.66	----------	[18]
**Graphene–Al–MoS_2_–Graphene**	MgF_2_	632.8	540.8	----------	[19]
**Air-MoS_2_–Al–MoS_2_–Graphene**	BK7	632.8	--------	8.19 × 10^4^	[39]
**Au–MoS_2_–Graphene**	BK7	632.8	----------	8.1852 × 10^4^	[20]
**Au–BlueP–Graphene**	SF11	632.8	70.0833	1.4731 × 10^5^	This work

## Supplementary Materials

The following are available online at https://www.mdpi.com/1424-8220/20/11/3326/s1. Figure S1: Change in differential phase with respect to the variation in local refractive index of sensing interface by modulating the numbers of BlueP interlayer and the thickness of Au film: (a) 40 nm, and (b) 45 nm, respectively. Table S1: The obtained phase detection sensitivity by slightly varying the SPR angle in an increment of ±0.01 degree.
