# Supplementary material for: Ultrasensitive Surface Plasmon Resonance Biosensor Using Blue Phosphorus–Graphene Architecture
Source: Sensors (Basel). 2020 Jun 11;20(11):3326. doi: 10.3390/s20113326 (PMC7308865; doi:10.3390/s20113326)
Supplement: Supplementary file 1 [file sensors-20-03326-s001.pdf]

Supplementary Materials

Ultrasensitive Surface Plasmon Resonance Biosensor Using Blue Phosphorus-Graphene Architecture

Keyi Li, Lintong Li, Nanlin Xu, Xiao Peng, Yingxin Zhou, Yufeng Yuan*, Jun Song and Junle Qu

Key Laboratory of Optoelectronic Devices and Systems of Ministry of Education and Guangdong Province, College of Physics and Optoelectronic Engineering, Shenzhen University, Shenzhen 518060, China; keyi_li07070023@163.com (K.L.); lintong_li@163.com (L.L.); nanlim_xu@163.com (N.X.); pengxiao_px@szu.edu.cn (X.P.); yls772@163.com (Y.Z); songjun@szu.edu.cn (J.S.); jlqu@szu.edu.cn (J.Q.)

* Correspondence: yfyuan@szu.edu.cn (Y.Y.); Tel.: +86- 0755-2690-9601

Supplementary Table

Table S1 The obtained phase detection sensitivity by slightly varying the SPR angle in an increment of ±0.01 degree

Angle shift	Incident angle	$\Delta\varphi_d$	Sensitivity (degree/RIU)
-0.10°	54.7152°	2.5686°	2.1405×10^3
-0.09°	54.7252°	2.5668°	2.1390×10^3
-0.08°	54.7352°	2.5649°	2.1374×10^3
-0.07°	54.7452°	2.5625°	2.1354×10^3
-0.06°	54.7552°	2.5591°	2.1326×10^3
-0.05°	54.7652°	2.5538°	2.1282×10^3
-0.04°	54.7752°	2.5441°	2.1201×10^3
-0.03°	54.7852°	2.5233°	2.1027×10^3
-0.02°	54.7952°	2.4661°	2.0551×10^3
-0.01°	54.8052°	2.1488°	1.7907×10^3
0.00°	54.8152°	176.7661°	1.4731×10^5
0.01°	54.8252°	177.1151°	1.4760×10^5
0.02°	54.8352°	177.1479°	1.4762×10^5
0.03°	54.8452°	177.1205°	1.4760×10^5
0.04°	54.8552°	177.0469°	1.4754×10^5
0.05°	54.8652°	176.9012°	1.4742×10^5
0.06°	54.8752°	176.5839°	1.4715×10^5
0.07°	54.8852°	175.5558°	1.4630×10^5
0.08°	54.8952°	23.5770°	1.9648×10^4
0.09°	54.9052°	0.6554°	5.4613×10^2
0.10°	54.9152°	1.4963°	1.2469×10^3

Supplementary Figure

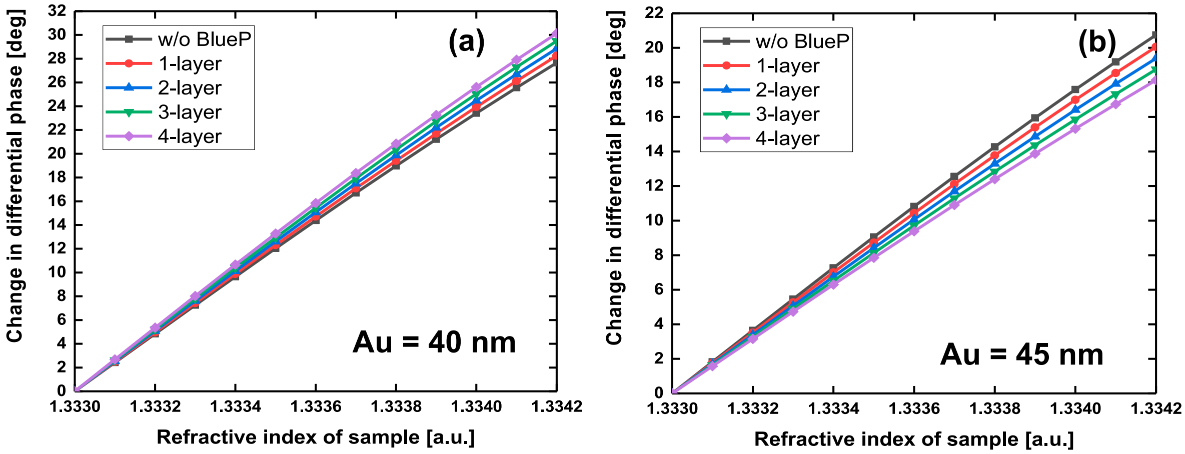

Figure S1. Change in differential phase with respect to the variation in local refractive index of sensing interface by modulating the numbers of BlueP interlayer and the thickness of Au film: (**a**) 40 nm, and (**b**) 45 nm, respectively.
